# Resilience Mitigates the Link between Adverse Childhood Experiences and Musician’s Dystonia: A Neuroendocrine and Psychological Perspective

**DOI:** 10.5334/tohm.1161

**Published:** 2026-03-13

**Authors:** Julian Burek, Stine Alpheis, Christopher Sinke, Tillmann H. C. Krüger, Michael Großbach, Daniel S. Scholz, Florian Worschech, André Lee, Eckart Altenmüller

**Affiliations:** 1 Institute of Music Physiology and Musicians’ Medicine, Hannover University of Music, Drama and Media, 30175 Hannover, Germany; 2 Hannover Medical School, 30625 Hannover, Germany; 3 Department of Musicians’ Health, University of Music Lübeck, 23552 Lübeck, Germany; 4 Institute of Medical Psychology, University of Lübeck, 23562 Lübeck, Germany; 5 Department of Psychiatry, Social Psychiatry and Psychotherapy, Hannover Medical School, 30625 Hannover, Germany; 6 Center for Systems Neuroscience, 30559 Hannover, Germany; 7 Department of Neurology, TUM Klinikum rechts der Isar, Munich, Germany

**Keywords:** Musician’s Dystonia, Resilience, Adverse Childhood Experiences, Cortisol, Stress Reactivity

## Abstract

**Background:**

Musician’s dystonia (MD) is a task-specific movement disorder affecting up to 1% of all professional musicians. Although adverse childhood experiences (ACEs) have been proposed as risk factors, the etiology of MD is not fully understood, and protective factors remain largely unexplored.

**Objective:**

This study investigated a possible protective role of psychological resilience on the association between MD and adverse childhood experiences and examined the influence of dystonia, resilience and ACEs on the stress reactivity of musicians.

**Methods:**

Forty participants with MD were compared to 39 matched healthy musicians. While undergoing the “Montreal Imaging Stress Task”, cortisol responses of the participants were measured. Furthermore, participants completed two psychological assessments, the Connor-Davidson Resilience Scale and the Childhood Trauma Questionnaire. Mediation and moderation analyses evaluated the mitigating role of resilience on the relationship between ACEs and MD. Bayesian multilevel models were used to analyze links between cortisol responses, ACEs and MD.

**Results:**

Healthy musicians showed higher resilience than MD patients, especially when looking at the dimensions “adaptability/flexibility” (*W* = 517, *p* = 0.004) and “regulation of emotion and cognition” (*W* = 554, *p* = 0.011). Resilience moderated the association between ACEs and dystonia (–0.29 [–0.47, –0.10]). MD patients and healthy participants did not differ in their cortisol output.

**Discussion:**

While links between acute stress reactivity, ACEs and MD were more equivocal, resilience seems to decrease the negative effects of ACEs on MD development.

The results of this study emphasize the need to implement resilience enhancing interventions at an early state of musical education.

**Highlights:**

Musicians suffering from musician’s dystonia (MD) and healthy musicians were compared concerning resilience, adverse childhood experiences (ACEs) and their stress reactivity.

Healthy musicians showed tendencies of higher resilience than MD patients.

Resilience appears to decrease the negative effects of ACEs on the development of musician’s dystonia.

MD patients and healthy musicians did not display differences in their cortisol output when presented with an acute stress task.

## 1. Introduction

Throughout the last decades, several studies have tried to identify the causes of and risk factors for focal task-specific dystonia. While neurological [1], genetic [2,3] and psychological [4,5,6] risk factors have been identified, little is known about protective factors that prevent the development of dystonia. One important protective factor for numerous disorders is resilience, the ability to maintain or restore normal functioning after being exposed to adverse events such as tragedy, trauma and illness [7,8].

Active coping, physical exercise, a supportive social environment and character traits such as optimism and humor can contribute to building resilience and stress tolerance and adapting to challenging life experiences [9,10,11]. In the past, several studies were conducted on the protective role of resilience in the health of individuals who had been exposed to significant life stressors such as adverse childhood experiences (ACEs) [13,14]. The term ACEs describes different forms of neglect and abuse as well as household dysfunctions that occurred to children and adolescents before the age of 18. ACEs are linked to chronic diseases, mental disorders and increased risk of injury [15]. They might also further contribute to the development of psychological disorders by impacting the hypothalamic-pituitary-adrenal (HPA) axis [16], one of our body’s main stress response systems. A study from 2020 showed that healthy young adults who experienced ACEs showed increased stress reactivity, exhibiting higher subjective stress and cortisol levels when confronted with psychosocial stressors in comparison to controls with no ACEs [17]. The effect of altered HPA-functioning on mental health disorders, e.g. depression, has received growing attention in recent years [18,19,20]. The literature has shown that resilience can mediate (i.e. explain the relationship between two variables) as well as moderate (i.e. impact the strength of) the association between ACEs and psychological health problems [13,21,22,23,24,25]. Further, research has shown that stress can modulate motor system function [26,27]. Psychological stress can lead to limb stiffness, more precisely to a higher level of co-contraction of agonist and antagonist muscles [28].

Resilience may thus be a particularly important protective factor against the development of musician’s dystonia (MD), since professional musicians are exposed to numerous psychological stressors throughout their career – e.g. performance pressure, high competitiveness, hours of extensive practicing, job insecurity [29,30,31,32,33] – and ACEs are hypothesized to increase MD risk by lowering stress resilience in this population [12].

Briefly, MD is a task-specific movement disorder characterized by muscle cramps and impaired voluntary motor-control during the playing of a musical instrument, impacting about 1% of professional musicians and leading to the premature termination of many professional careers [35,36,37]. While MD is typically classified as a neurological disorder, there is an ongoing discussion about psychological components in the etiology of the disorder [38].

It is possible that ACEs are a risk factor for developing MD by affecting the musicians’ mental health as well as increasing their stress reactivity through alterations in HPA-functioning.

The current study aimed to investigate the role of resilience in MD patients and in particular the possible protective role of resilience as a mediator/moderator of the relationship between ACEs and MD.

We therefore formulated the following hypotheses: a) MD patients have lower resilience levels compared to healthy controls, b) resilience has both a mediating as well as a moderating effect on the association between ACEs and dystonia and c) resilience, ACEs and dystonia influence the individual neurobiological stress reactivity of musicians, measured through the cortisol output during a social evaluative stress task.

## 2. Material and Methods

### 2.1 Participants

This paper is part of a larger study investigating risk factors of MD, the procedures of which are described in more detail elsewhere [39]. Seventy-nine professional musicians participated in the study. Forty of the participants (age 47.9 ± 10 years, 14 female) were diagnosed with MD of the upper extremity (fingers, hands) and recruited via the patient database of the Institute of Music Physiology and Musicians’ Medicine, Hannover. As a control group, 39 healthy musicians (HM; age 49.2 ± 11.9 years, 14 female) were recruited via public appeals (flyers, social-media advertisements) and by contacting Northern German orchestras and the staff and students of the University of Music, Drama and Media, Hannover. MD and HM group were matched as best as possible in terms of age, sex, and instrument. If no suitable control participants could be identified for specific MD patients using the procedures described above, all participants (MD as well as controls) were asked to mobilize their professional and personal networks to recruit healthy musicians who met the predefined matching criteria. Participant details can be found in Table 1.

**Table 1:** Descriptive characteristics of participants.


PARAMETER	MD (n = 40) *M (±SD)*	HM (n = 39) *M (±SD)*	TEST STATISTIC *W*	*p-value*

Age, y	47.93 (10.02)	49.15 (11.92)	708	0.483

Sex, male/female/divers (n)	26/14/0	25/14/0	χ^2^ (1, N = 79) = 0.007	0.934

Age started playing the instrument, y	9.68 (4.91)	4.74 (17.29)	523	0.011*

Instrument group, n (%)				

Keyboard	10 (25%)	10 (25.6%)		

String instruments	8 (20%)	9 (23.07%)		

Woodwind instruments	13 (32.5%)	13 (33.33%)		

Plucked	9 (22.5%)	7 (17.95%)		

Dystonia age at onset, y	33.63 (9.83)			


*Note*. Test statistics show *W* of the Wilcoxon rank-sum test unless otherwise indicated. *p < 0.05. MD = musician’s dystonia patients; HM = healthy musician; M = mean; SD = standard deviation. This information is reprinted in part from Alpheis et al. [39] under the terms of the Creative Commons Attribution Non-Commercial Share-Alike (CC BY-NC-SA) 4.0 International License.

All participants were either studying music or working as music teachers, soloists, freelancers or in orchestras. Exclusion criteria were pregnancy, severe visual deficits, any other form of movement disorder or suffering from extensive brain injury. Since many MD patients take medication (e.g. Trihexyphenidyl), it was decided not to apply current medication as an exclusion criterium, as this would have reduced the sample size considerably. All participants received monetary compensation (60€) and full travel reimbursement for their participation. They gave written informed consent prior to the start of the study and were free to withdraw at any time. The data were collected between April 2022 and April 2023 at facilities of Hannover Medical School. The study design was approved by the local ethics committee of Hannover Medical School (No. 9914_BO_S_2021).

### 2.2 Assessment of resilience

Resilience was assessed using the Connor-Davidson Resilience Scale (CD-RISC) [40,41]. The CD-RISC is a questionnaire containing 25 items. It uses a five-point Likert scale, ranging from 0 to 4. Higher scores indicate higher resilience. The CD-RISC consists of different statements, describing different aspects of resilience: hardiness, coping, adaptability/flexibility, meaningfulness/purpose, optimism, regulation of emotion and cognition, and self-efficacy. According to the authors, these are not to be considered as sub-scales but merely reflect different dimensions of resilience. Three participants answered this and the following questionnaire (section 2.3) in English, with all other participants responding to the German language versions.

### 2.3 Assessment of adverse childhood experiences

ACEs were assessed using the Childhood Trauma Questionnaire (CTQ) [42,43]. The CTQ is a 28-item screening tool which assesses emotional, physical, and sexual abuse, as well as emotional and physical neglect on a five-point Likert scale. Higher CTQ scores reflect more or more severe ACEs.

### 2.4 Stress induction using the Montreal Imaging Stress Task

To enable investigation of the associations between resilience, ACEs and acute stress reactivity, all participants were subjected to the Montreal Imaging Stress Task (MIST) in addition to answering questionnaires. The MIST [44] is a tool developed to induce and examine the effects of psychosocial evaluative stress. The MIST was chosen because it is designed to be used during functional magnetic resonance imaging (fMRI). The neurological stress reactions were also of interest for the overall research question of this project.

**Figure 1:**
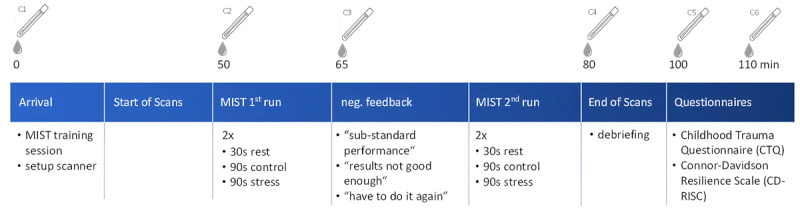
Study design. The participants completed two runs of the Montreal Imaging Stress Task (MIST) during functional magnetic resonance imaging (fMRI). Afterwards, all participants completed psychological questionnaires. Saliva samples were taken at six different timepoints, at 0, 50, 65, 80, 100 and 110 minutes (C1–C6). This information is reprinted in part from Alpheis et al. (2024) under the terms of the Creative Commons Attribution Non-Commercial Share-Alike (CC BY-NC-SA) 4.0 International License.

The MIST paradigm has three test conditions: rest (30 secs), control (90 secs) and experimental (90 secs; see Figure 1 and Figure S1 in the supplements).

While during the rest condition no task was presented to the participants, in the subsequent control (without time pressure) and stress condition (with time pressure), participants were asked to solve mathematic equations of varying difficulties which appeared on the screen during fMRI.

Furthermore, after every answer, participants received visual feedback on whether they solved the equation correctly. An additional bar at the top of the screen showed that the subject’s performance was below average in comparison to the other participants of the study (even if this was not true).

In total, all participants had to complete two runs of the MIST. After the first run, the instructor of the experiment gave the participants scripted negative feedback, saying that their performance was subpar, that the data were not comparable and therefore could not be used for the analysis. They informed the participants that the task had to be repeated and asked them to concentrate more to achieve better results. Cortisol release was measured before the experiment, between the two runs, and after the MIST (see Figure 1 for full study design).

To blind the participants from the actual objective of the MIST – inducing stress – they were told that the task would be used to learn about differences in dystonia patients and healthy musicians concerning their brain activity during a resting state and a state of concentration. After completion of the task all participants were thoroughly debriefed. The neurological data gained during fMRI are presented in a separate paper [39].

### 2.5 Assessment of cortisol

Salivary cortisol was collected at baseline and after 50, 65, 80, 100 and 110 minutes of the experiment using a Salivette (Sarstedt, Nümbrecht, Germany). Upon arrival, participants had a fifteen-minute rest and were informed about the procedure of the experiment before the first sample (t = 0) was taken. Three further samples were taken directly before (t = 50), between (t = 65) and directly after (t = 80) the two MIST runs. The final two samples were collected twenty (t = 100) and thirty minutes (t = 110) after the second MIST run. All measurements took place in the afternoon between 4.00–7.00 pm to account for circadian fluctuations of cortisol [16,45]. The participants were instructed not to eat or drink anything except water for one hour before the start of the experiment. Furthermore, they were asked not to drink alcohol, use drugs, consume unusual amounts of caffeine/nicotine, or exercise heavily for the 12 hours prior to the experiment. Women who were not already in menopause were tested in the second phase of their menstrual cycle (at least 14 days after the beginning of their last period).

The samples were analyzed using electrochemiluminescence immunoassay (ECLIA) in an external lab (*amedes MVZ wagnerstibbe für Laboratoriumsmedizin;* https://www.wagnerstibbe-hannover.de). Here, Elecsys Cortisol II cobas e 801 analytical units were used to measure cortisol. According to the product description, the limit of detection (i.e. the lowest detectable analyte concentration) was 0.54 µg/l; the limit of quantitation (i.e. the lowest amount of analyte that can be accurately quantitated with an error of ≤30%) was 1.09 µg/l. Since therefore the exact amount of cortisol for concentration levels below 1.1 µg/l could not be specified but was known to be between 0.54 and 1.08 µg/l, an average concentration of 0.81 µg/l was assumed for further analysis of values falling below this threshold. For the analysis, the precision of the quantitation method (i.e. the measurement error), which is known from the product specifications, was taken into account for each probe. The magnitude of the error depended on the cortisol concentration (see supplements Table S1).

Three participants (2 HM, 1 MD) were excluded from the cortisol analysis; one participant could not complete the stress task due to unexpected visual problems, two other participants experienced either a panic attack within the MRI or reported a severe headache which ultimately led to extraordinarily high cortisol levels.

### 2.6 Statistical analysis

Statistical analyses were performed in R [46]. Questionnaire data from CD-RISC were compared between groups using one-sided Wilcoxon rank-sum tests, since not all data were normally distributed. Before regression analyses, multicollinearity checks were performed. Although we reported the exact *p*-value, we intentionally did not specify a significance level. Instead, we followed the American Statistical Association’s (ASA) statement on statistical significance, which emphasizes that *p*-values are neither a measure of evidence nor an indication of whether an effect is true or not [47].

The influence of CD-RISC, CTQ, and MD on cortisol were analyzed with Bayesian multilevel models using the package “brms” [48]. We selected a Weibull family as the response distribution. To compute cortisol changes for each measurement time point, we applied linear splines. Individual measurement errors of the probes were incorporated in the model as known uncertainties using the function mi(). We specified only regulating noninformative priors.

Three independent models were specified with CD-RISC, CTQ and MD as predictors. Before, MD was dummy-coded and centered, which references the population effects to the “average” sample. CD-RISC and CTQ were *z*-transformed and their effects on cortisol are presented in the results as a contrast between “low” (0.5 SD below mean) and “high” (0.5 SD above mean). Participants’ sex was also dummy-coded and centered and included as a covariate in each model. Time was coded in hours (0, 0.833, 1.083, 1.333, 1.667, 1.833) and allowed to interact with CD-RISC, CTQ and MD.

The association between CD-RISC, CTQ and MD was tested among competing Bayesian models. CD-RISC was examined as a mediator, moderator or both within the relationship between CTQ and MD.

In a secondary post-hoc analysis, additional CTQ-data from a previous study of our working group [49] was added to the statistical analysis (“Bayesian Information Borrowing”). The study included 114 MD patients and 134 HMs and examined the relationship between musician’s dystonia and adverse childhood experiences using the CTQ. The inclusion of this data substantially increased our sample size and improved statistical power. This enabled a more accurate estimation of the relationship between CTQ responses and MD.

After comparing all models using expected log pointwise predictive densities (elpd), the best model was selected. All models were run with 10,000 iterations, including a warm-up of 5,000 iterations. Results are presented with 95% credible interval and model fit was verified by posterior-predictive checks. Convergence was estimated by Rhat-values and visual inspection of the Markov chains. All models converged without any problems, showing no divergent transitions.

## 3. Results

### 3.1 Psychological questionnaire data

According to the CD-RISC_total_ score, MD patients tended to be less resilient than healthy participants (*W* = 627, *p* = 0.067; Table 2). MD patients showed lower scores on the dimensions “adaptability/flexibility” (*W* = 517, *p* = 0.004; Table 2) and “regulation of emotion and cognition” (*W* = 554, *p* = 0.011; Table 2).

**Table 2:** Results and group comparison of the Connor-Davidson Resilience Scale (CD-RISC).


PARAMETER	MD (n = 40) MDN (±IQR)	HM (n = 39) MDN (±IQR)	TEST STATISTIC *W*	*p-VALUE*	EFFECT SIZE

CD-RISC total score	67.5 (20.0)	73.0 (12.0)	627	.067	0.197

Hardiness	21.5 (7.00)	22.0 (5.00)	734	.325	0.060

Coping	15.0 (6.00)	15.0 (2.00)	706	.233	0.095

Adaptability/Flexibility	9.00 (2.25)	10.0 (2.00)	517	.004*	0.338

Meaningfulness	9.00 (6.00)	10.0 (2.50)	718	.272	0.079

Optimism	5.00 (2.00)	5.00 (1.00)	639	.076	0.181

Reg. of emotion and cognition	5.50 (1.50)	6.00 (1.00)	554	.011*	0.290

Self-efficacy	6.00 (3.00)	6.00 (1.00)	779	.506	0.001


*Note*. Test statistics show *W* of the Wilcoxon rank-sum test. *p < 0.05. MD = musician’s dystonia patients; HM = healthy musicians; MDN = median; IQR = interquartile range; CD-RISC = Connor-Davidson Resilience Scale; Reg. of emotion and cognition = Regulation of emotion and cognition.

MD patients (Mdn = 33.50; IQR = 16.00) reported more ACEs compared to healthy musicians (Mdn = 29.00; IQR = 10.00) reflected by a higher CTQ_total_ score (*W* = 581, *p* = 0.025).

### 3.2 Cortisol & CD-RISC

**Figure 2:**
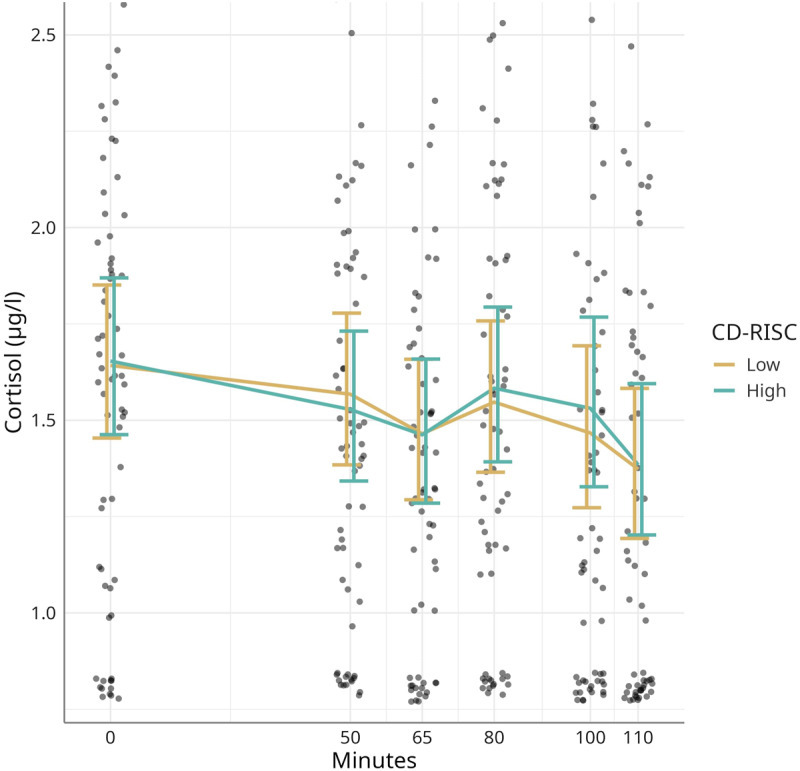
The effect of CD-RISC_total_ scores on cortisol responses during the experiment. Modelled means with 95% credible intervals and observed data points. CD-RISC = Connor-Davidson Resilience Scale.

The cortisol concentrations changed throughout the experiment: as shown in Figure 2, during the first 50 minutes, there was a possible slight decrease (–0.07, 95% credible interval [–0.16, 0.01]), followed by a stronger decrease (–0.22 [–0.38, –0.06]) in cortisol concentration. During minutes 65 to 80, there was a strong increase (0.27 [0.05, 0.49]). At the end of the experiment, there was a possible slight decrease in cortisol (80–100 minutes: –0.13 [–0.32, 0.07]), followed by a strong decrease (100–110 minutes: –0.50 [–0.67, –0.32]).

Women showed lower cortisol concentrations in comparison to men (–0.23 [–0.41, –0.04]). Higher CD-RISC_total_ scores indicating greater resilience were associated with a stronger cortisol decline during the last period of the assessment (–0.20 [–0.39, –0.02]). No other effects were found.

### 3.3 Cortisol and the Childhood Trauma Questionnaire

**Figure 3:**
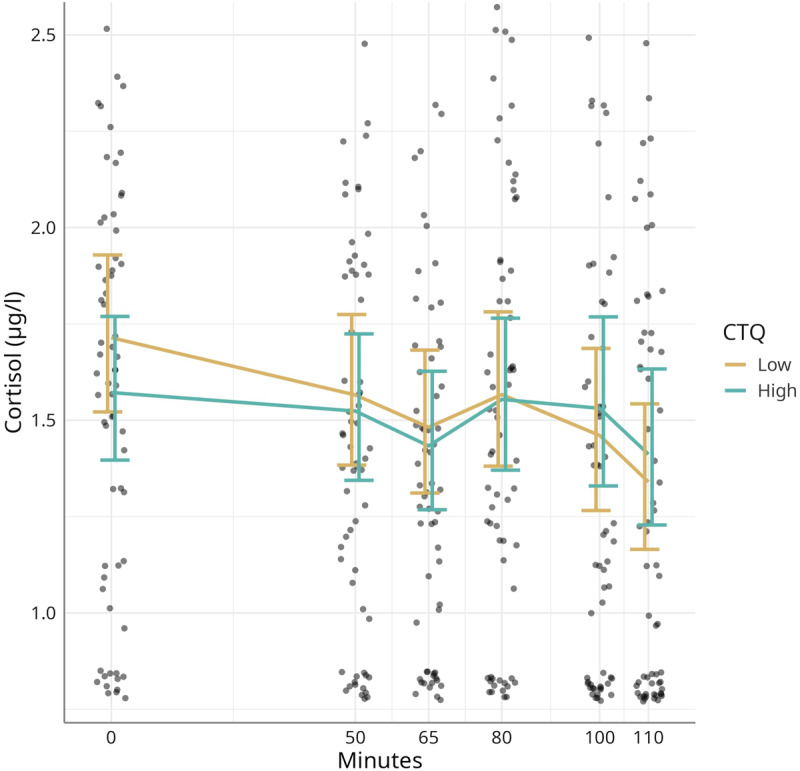
The effect of CTQ_total_ scores on the cortisol response during the experiment. Modelled means with 95% credible intervals and observed data points. CTQ = Childhood Trauma Questionnaire.

The links between cortisol responses and ACEs as reflected by CTQ scores were inconclusive. As portrayed in Figure 3a higher CTQ_total_ score was likely associated with lower baseline cortisol concentration (–0.08 [–0.18, 0.02]) and a less pronounced cortisol decline from minutes 0–50 (0.07 [–0.02, 0.16]) and from minutes 80–100 (0.17 [–0.03, 0.36]).

### 3.4 Cortisol and Musician’s dystonia

Contrary to our hypothesis, neither cortisol concentrations nor cortisol changes differed between MD and HM in any study period.

### 3.5 Mediation and moderation models

**Figure 4:**
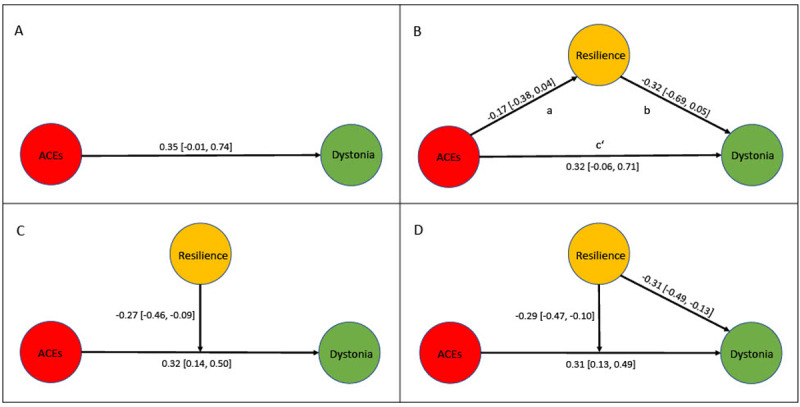
Hypothetical casual models to explore the association between ACEs (CTQ_total_ score) and dystonia as well as the mediating and moderating effect of resilience (CD-RISC_total_ score) on this relationship. **(A)** The direct effect of ACEs on dystonia. **(B)** Mediation analysis model: the mediating effect of resilience on the association of ACEs and dystonia. **(C)** Moderation analysis model: the moderating effect of resilience on the association of ACEs and dystonia. **(D)** Final model: the moderating effect of resilience on the association of ACEs and dystonia and its direct effect on dystonia. ACEs = Adverse childhood experiences; CTQ = Childhood Trauma Questionnaire; CD-RISC = Connor-Davidson Resilience Scale.

Logistic regression was used to predict the relationship between adverse childhood experiences and the development of MD (Figure 4, Model “A”). In the present sample (n = 79) musicians with higher CTQ scores were more likely to have MD than musicians who score low on the CTQ (OR = 1.42 [0.99, 2.09]).

This relationship was corroborated in an extended sample including data from Alpheis et al. [49] (total sample size n = 327), the OR was 1.55 [1.26, 1.96].

A mediation analysis (Figure 4, Model “B”) was conducted to assess a possible mediating effect of resilience on the relationship between ACEs (independent variable) and MD (dependent variable). We did not find a meaningful effect of ACEs on resilience (Model “B”, Pathway “a”, (–0.17 [–0.38, 0.04])). Thus, we rejected the mediation model.

In Model “C” (Figure 4), resilience was used as a moderating variable to determine its possible moderating role between ACEs and MD. ACEs were positively associated with dystonia (0.32 [0.14, 0.50]) and, moreover, resilience moderated the relationship between ACEs and MD (–0.27 [–0.46, –0.09]).

In Model “D” (Figure 4), we conducted a moderation model, investigating both the moderating effect resilience has on the relationship between ACEs and MD and the direct effect resilience has on the development of MD.

After comparing all models using expected log pointwise predictive densities (elpd; [50]), Model “D” showed the best fit. According to this model, ACEs were positively associated with dystonia (0.31 [0.13, 0.49]), whereas increased individual resilience negatively related to the development of MD (–0.31 [–0.49, –0.13]). Furthermore, resilience moderated the relationship between ACEs and MD (–0.29 [–0.47, –0.10]).

## 4. Discussion

The present study aimed to investigate the relationship between adverse childhood experiences, resilience and musician’s dystonia. To our knowledge, this is one of the largest samples of musicians with and without focal hand dystonia to have been compared with respect to cortisol release during acute stress.

Our results suggest that healthy musicians tend to be more resilient than MD patients in specific domains. Furthermore, resilience appears to moderate the relationship between ACEs and MD. Although MD patients did not differ from healthy musicians in their cortisol output, more resilient participants (higher CD-RISC_total_ scores) showed a stronger cortisol decline after the stress task was finished. Greater ACEs (higher CTQ_total_ score) were likely associated with a lower baseline cortisol concentration.

ACEs do appear to be associated with the development of MD. As confirmed by a post-hoc analysis of an extended sample of 327 musicians, experiencing more ACEs (defined as 0.5 SD above the mean of the CTQ) seemingly increases the risk of developing MD by about 50%. As mentioned above, ACEs are linked to increased prevalence of anxiety disorders, mood disorders, and certain character traits (e.g. neuroticism) that have also been observed in MD patients [6,51,52]. These traits, as well as social phobia, were identified as psychological predispositions which make musicians more likely to develop MD [5,6]. Resilience appears to moderate the association between ACEs and MD, however, due to the cross-sectional design of the study, the direction of this association is difficult to deduce. It is in any case plausible that higher resilience has a mitigating effect on the lasting negative consequences of ACEs. Other studies have proposed that high resilience prevents the onset of disease and that there is an anti-inverse relationship between resilience and illness: the higher the resilience, the lower the vulnerability and disease risk [7].

Although the authors of the CD-RISC questionnaire do not recommend an interpretation of the subdomains as actual subscales, a look at the subdomains of the CD-RISC questionnaire may provide some clues as to how resilience may moderate this relationship to guide further research [41]. MD patients scored lower on the dimensions “adaptability/flexibility” (*W* = 517, *p* = 0.004) and “regulation of emotion and cognition” (*W* = 554, *p* = 0.011). This could indicate that, in the face of an acute stressor, MD patients are less able to react and adapt flexibly and to regulate their emotions in a functional way. Since first symptoms of MD often occur during life periods of increased stress exposure, musicians who are less able to cope with these stressors could be more prone to suffering from stress induced movement disorders such as MD [35,53]. A lack of adaptability and flexibility may also translate to dysfunctional repetitive practice behaviors in these musicians, which have been suggested to increase playing related injuries and overuse syndromes which are often present at the beginning of dystonia [54]. Resilience enhancing methods should therefore receive more attention in music education. A systematic review from 2025 identified several psychological and physical interventions, including Cognitive Behavioral Therapy (CBT), Acceptance and Commitment Therapy, mindfulness and yoga, which increased the resilience levels of musicians [55]. These findings underline the fact that resilience can be modified. Since resilience seems to play a protective role in the pathophysiology of MD, especially of musicians who have been exposed to ACEs, music teachers as well as parents and others who are part of the musical education of children should be informed about the importance of resilience and incorporate resilience enhancing methods in their teaching.

Surprisingly, MD and HM groups did not differ in their neurobiological stress reactivity, as captured by cortisol release during the acutely stressful Montreal Imaging Stress Task. This could be due to the fact that the stressor was equally new to all of them and that, as musicians, their neurological system is accustomed to psycho-socially stressful events. A third control group of healthy non-musicians would be needed in future studies to confirm this hypothesis. It must further be acknowledged that the MIST has been developed to induce acute stress and does not represent the chronic stress the participants have possibly been exposed to throughout their life span. Future research could try to evaluate HPA activity of HM and MD patients over a prolonged period of time to discover possible differences in their cortisol output when exposed to daily stressors.

When looking at the association between resilience scores and cortisol release, higher CD-RISC_total_ scores were associated with a stronger cortisol decline during the last period of the assessment.

One explanation for these findings may be that individuals possessing higher levels of resilience were able to recover more quickly from stressful situations. As professional musicians often deal with extremely stressful working conditions which leads to constant activation of the HPA axis, musicians high in resilience would benefit from quicker recovery from stress. Elevated cortisol levels over an extended period are linked to various mental and physical health issues such as depression, cardiovascular diseases, and cancer [56,57,58,59]. Besides, as already reported, psychological stress can affect motor system function [28,34], in particular muscle tension and limb stiffness, a hallmark of MD [60,61]. It can thus be hypothesized that resilience can protect musicians from developing movement disorders such as MD by enhancing the ability to recover more quickly from stressful events, not only from a mental, but also from a neuroendocrinological standpoint.

Another possible explanation could be that the cortisol decline in individuals with higher CD-RISC_total_ scores is greater due to a preceding elevated cortisol output, especially during the minutes 65–70 (see Figure 2). However, the cortisol decline between minutes 100–110 is not correlated with the cortisol incline during the period of maximum stress induction (min. 65–80), nor is the decline correlated with any other cortisol changes during the study.

We further expected musicians showing higher rates of ACEs to be more vulnerable to stress (as measured by cortisol release). In contrast to our hypothesis, participants with higher CTQ scores did not show an increased HPA axis activity but rather lower baseline cortisol concentrations. The literature is inconsistent when looking at the exact impact ACEs have on HPA axis functioning. A study from 2020 investigated the possible impact of a dysfunctional HPA axis on the relationship between childhood maltreatment and depression [17]. On the one hand, they showed that individuals who have been exposed to early adversity reacted more sensitively when being confronted with a psychosocial stressor, exhibiting increased cortisol levels in comparison to controls who have not faced any childhood maltreatment. On the other hand, there are several studies reporting diminished cortisol responses to stress when examining adults who experienced ACEs [62,63,64].

Basal hypocortisolism despite acute stress could be explained by chronic glucocorticoid hypersecretion exhibited while being exposed to chronic, sustained stress early in life [65]. This chronic cortisol hypersecretion could then lead to a downregulation of corticotropin-releasing factor (CRF) receptors and increased negative glucocorticoid feedback [65].

### 4.1 Limitations

Our study results must be considered in the context of several limitations.

Firstly, as already discussed above, resilience is a broad term which includes various internal and external factors. The CD-RISC questionnaire can differentiate between people with high and low resilience but only gives little information as to which specific aspects of resilience were most salient. Therefore, our findings regarding the mechanisms with which resilience might influence the association of ACEs and MD should be interpreted with caution.

Secondly, since we conducted a cross-sectional study with retrospective information about ACEs, it is difficult to determine causality in the links between resilience, ACEs and MD with absolute certainty. We found a moderating effect of resilience on the association between ACEs and MD, but we do not know if some participants were simply born with a resilient predisposition or acquired resilience later in life, during or after experiencing ACEs. More research is needed to discover when and how resilience moderates the negative impact of ACEs on dystonia to implement resilience-enhancing interventions in future young musicians.

Thirdly, we did not assess the duration, number of ACEs, nor age of the participants when exposed to childhood adversity. All these components could potentially lead to highly different outcomes concerning the CTQ and HPA axis activity results. Previous studies showed [63], for example, that participants with high exposure to ACEs demonstrated a significantly blunted cortisol response when confronted with a psychosocial stress task in comparison to a control group with lower ACE exposure. Furthermore, memory biases can lead to underreporting and overreporting of actual adverse experiences when looking at the CTQ results [66].

Lastly, cortisol as a biomarker of psychological stress and its production is highly individual and can be influenced by several confounders which were, in principle, controlled for (sex, age, menstrual cycle). However, individual variability in cortisol levels could have nonetheless significantly influenced our results. Additionally, as mentioned above, there were difficulties to accurately detect cortisol concentrations below 1.1 µg/l due to the limit of quantitation of the ECLIA used to analyze the saliva samples.

## 5. Conclusion

As part of a larger study investigating risk factors of musician’s dystonia, this paper is to our knowledge among the first investigations to explore the relationship between resilience, childhood adversities and stress reactivity in MD patients. Our findings indicate that healthy musicians tend to be more resilient than MD patients in some domains. Moreover, resilience seems to decrease the negative effects of adverse childhood experiences on the development of MD. Links between acute stress reactivity, ACEs, resilience and MD were more equivocal. Future research is needed which further explores the psychological aspects of MD, as well as resilience training as a possible protective intervention for MD.

## Data Accessibility Statement

Data supporting the findings of this study are available from the corresponding author upon reasonable request.

## Additional Files

The additional files for this article can be found as follows:

10.5334/tohm.1161.s1 Figure S1. Examples of the mathematic equations participants were confronted with during the Montreal Imaging Stress Task (MIST).

10.5334/tohm.1161.s2 Table S1. Cortisol concentrations and their respective measurement errors.
