# Supplementary material for: Resilience Mitigates the Link between Adverse Childhood Experiences and Musician’s Dystonia: A Neuroendocrine and Psychological Perspective
Source: Tremor Other Hyperkinet Mov (N Y). 2026 Mar 13;16:16. doi: 10.5334/tohm.1161 (PMC12985811; doi:10.5334/tohm.1161)
Supplement: Figure S1. — Examples of the mathematic equations participants were confronted with during the Montreal Imaging Stress Task (MIST). [file tohm-16-1-1161-s1.pdf]

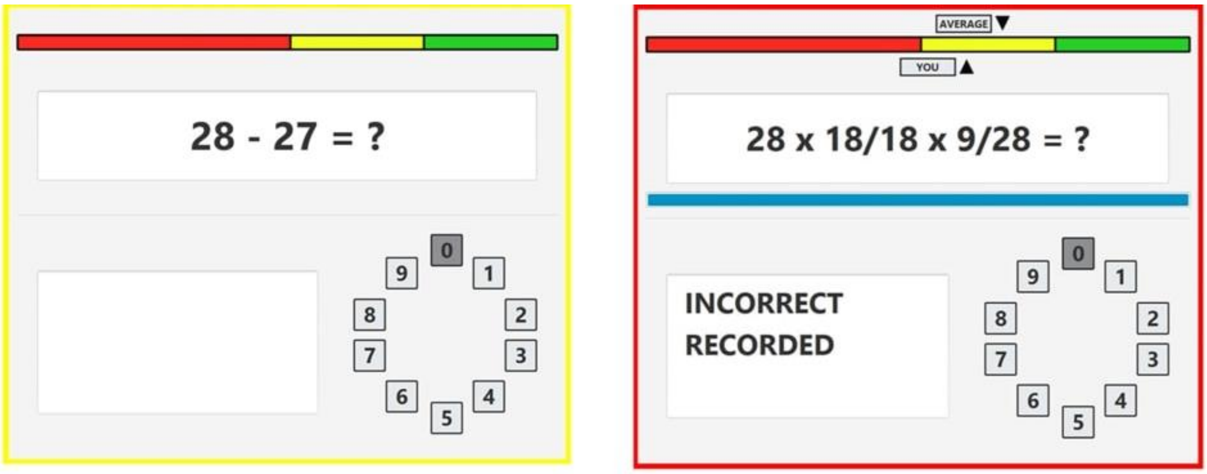

Figure S1: Examples of the mathematic equations participants were confronted with during the Montreal Imaging Stress Task (MIST).

ALT zu Fig. S1 (supplements): Mathematic equations shown during the Montreal Imaging Stress Task (MIST).
