# Supplementary material for: Resilience Mitigates the Link between Adverse Childhood Experiences and Musician’s Dystonia: A Neuroendocrine and Psychological Perspective
Source: Tremor Other Hyperkinet Mov (N Y). 2026 Mar 13;16:16. doi: 10.5334/tohm.1161 (PMC12985811; doi:10.5334/tohm.1161)
Supplement: Table S1. — Cortisol concentrations and their respective measurement errors. [file tohm-16-1-1161-s2.pdf]

**Table S1**

**Cortisol concentrations and their respective measurement errors.**

| Cortisol concentration (µg/l) | Measurement error (SD) |
|---|---|
| 8.99 – 10.8 | 0.13 |
| 7.18 – 8.98 | 0.115 |
| 5.36 – 7.17 | 0.1 |
| 3.54 – 5.35 | 0.08 |
| 2.45 – 3.53 | 0.065 |
| 1.18 – 2.44 | 0.05 |
| 1.09 – 1.17 | 0.04 |
| 0.54 – 1.08 (= 0.81) | 0.06 |

*Note.* SD = standard deviation
